# Expanding the miRNA Repertoire in Atlantic Salmon; Discovery of IsomiRs and miRNAs Highly Expressed in Different Tissues and Developmental Stages

**DOI:** 10.3390/cells8010042

**Published:** 2019-01-11

**Authors:** Nardos Tesfaye Woldemariam, Oleg Agafonov, Bjørn Høyheim, Ross D. Houston, John B. Taggart, Rune Andreassen

**Affiliations:** 1 Department of Life Sciences and Health, Faculty of Health Sciences, OsloMet–Oslo Metropolitan University, Pilestredet 50, N-0130 Oslo, Norway; nate@oslomet.no; 2 Bioinformatics Core Facility, Department of Core Facilities, Institute of Cancer Research, Radium Hospital, Oslo University Hospital, 0379 Oslo, Norway; olegag@ifi.uio.no; 3 Department of Basic Sciences and Aquatic Medicine, Faculty of Veterinary Medicine, Norwegian University of Life Sciences, 0454 Oslo, Norway; bjorn.hoyheim@nmbu.no; 4 Division of Genetics and Genomics, The Roslin Institute and Royal (Dick) School of Veterinary Studies, University of Edinburgh, Midlothian EH25 9RG, UK; ross.houston@roslin.ed.ac.uk; 5 Institute of Aquaculture, University of Stirling, Stirling, FK9 4LA, UK; j.b.taggart@stir.ac.uk

**Keywords:** Teleostei, embryogenesis, tissue-enriched miRNAs, post-transcriptional gene regulation

## Abstract

MicroRNAs (miRNAs) are important post-transcriptional gene expression regulators. Here, 448 different miRNA genes, including 17 novel miRNAs, encoding for 589 mature Atlantic salmon miRNAs were identified after sequencing 111 samples (fry, pathogen challenged fry, various developmental and adult tissues). This increased the reference miRNAome with almost one hundred genes. Prior to isomiR characterization (mature miRNA variants), the proportion of erroneous sequence variants (ESVs) arising in the analysis pipeline was assessed. The ESVs were biased towards 5’ and 3’ end of reads in unexpectedly high proportions indicating that measurements of ESVs rather than Phred score should be used to avoid misinterpreting ESVs as isomiRs. Forty-three isomiRs were subsequently discovered. The biological effect of the isomiRs measured as increases in target diversity was small (<3%). Five miRNA genes showed allelic variation that had a large impact on target gene diversity if present in the seed. Twenty-one miRNAs were ubiquitously expressed while 31 miRNAs showed predominant expression in one or few tissues, indicating housekeeping or tissue specific functions, respectively. The miR-10 family, known to target *Hox* genes, were highly expressed in the developmental stages. The proportion of miR-430 family members, participating in maternal RNA clearance, was high at the earliest developmental stage.

## 1. Introduction

MicroRNAs (miRNAs) are short non-coding RNAs that play an important role in post-transcriptional regulation of gene expression [1]. After transcription, the large primary miRNA transcripts (pri-miRNAs) are cleaved into shorter miRNA precursors (pre-miRNAs) that are exported out of the nucleus by Exportin 5. Here they are processed further by Dicer to produce the mature 5p and 3p miRNAs that are about 20–24 nts long. The mature miRNAs are then loaded onto Argonaute proteins and incorporated in the RNA induced silencing complex (miRISC). As part of the miRISC they form partially complementary bindings with their target mRNAs which subsequently leads to degradation or translational repression of the target transcripts [1,2]. The characteristics of these miRNA precursors and the mature miRNAs produced from Dicer cleavage of the miRNA precursors may be utilized to design bioinformatics tools that identify the miRNA genes and their mature miRNAs. High-throughput sequencing of small RNAs that are analyzed with such dedicated bioinformatics tools against a reference genome allows for massive parallel identification of a large number of miRNA genes [3,4].

Several studies have demonstrated that miRNAs are involved in many biological processes such as development, growth, tissue differentiation and apoptosis [5,6]. There is also evidence that some miRNAs are important regulators of immune function and immune responses [7,8]. As their biological function is defined by the sequence of the mature miRNAs, characterization of the miRNA repertoire is a first step to understand how miRNAs participate in the regulation of a species gene networks. 

Genomic research in Atlantic salmon (*Salmo salar*) has been carried out due to the cultural and recreational importance of this species, and more importantly, its economic importance as an aquaculture species (www.fao.org/fishery/affris/species-profiles/atlantic-salmon/atlantic-salmon-home/en/). Atlantic salmon miRNAs were first characterized in 2013 by Andreassen et al. [9] and Bekaert et al. [10] utilizing the first assembly of the Atlantic salmon genome sequence. These miRNA reference sequences (miRNAome) has been used to develop RT-qPCR methods to measure miRNA expression, to investigate the role of miRNAs in host-virus responses, miRNAs that may affect sea louse infestation and miRNAs that may affect testis development [11,12,13,14]. 

The development of sequencing technology and the increased sensitivity of deep sequencing has led to the discovery of isomiRs [15,16,17]. IsomiRs are mature miRNA sequence variants with different 5’ and/or 3’ ends compared to their corresponding canonical mature miRNAs. IsomiRs are assumed to be products of imprecise cleavage of pre-miRNAs, RNA editing and non-templated nucleotide additions at the 3′ end of miRNAs [17,18]. Knowledge about the function of isomiRs is limited, but it has been suggested that isomiRs cooperate with their corresponding canonical miRNAs to target common biological pathways. Any post-transcriptional modification of the 5’ end of a mature miRNA (5’ isomiRs) is of particular importance as such changes affect the “seed” sequences. Any change of “seed” would affect target specificity, and by this, potentially increase the number of transcripts targeted [18,19]. 

Small-RNA sequencing datasets consist of a huge number of reads. Some differences in length or sequence among reads are due to errors arising in the sequencing pipeline like RNA degradation, sequence errors introduced in cDNA synthesis or during sequencing [20,21]. Such artefacts may be misinterpreted as isomiRs. Therefore, to characterize isomiRs that are true products of biological post-transcriptional processing one need to distinguish isomiRs from such artefacts arising in the deep sequencing pipeline. 

So far, there have been no studies to characterize isomiRs in Atlantic salmon. Although the first miRNA discovery studies provided an important species-specific reference [9,10], the materials sequenced were from relative few samples. No samples from different developmental stages or tissues from fish challenged with pathogens were investigated. 

In the present study, about ten times more samples have been included compared to the initial study [9]. One hundred and eleven small RNA libraries from different tissues sampled from adult fish, from different developmental stages, from normal fry, as well as from fry challenged with pathogens were sequenced. Thus, the materials investigated has allowed us to identify miRNAs likely to have tissue and developmental stage specific functions. The proportion of isomiR-like artifacts generated in the small-RNA sequencing pipeline was also revealed. On this background, the first characterization of isomiRs in Atlantic salmon was carried out. Nearly one hundred new miRNA genes, both miRNAs conserved in teleosts, as well as novel miRNAs, were discovered. The new reference miRNAome, where miRNA gene locations are assigned to the present genome sequence [22], forms an important updated resource for miRNA expression studies, as well as for comparative studies of miRNA gene evolution. 

## 2. Materials and Methods

### 2.1. Materials

A total of 111 samples were sequenced in this study. Samples 1–96 in Table S1 were from fry, while samples 97–111 were tissue samples from particular organs from fully developed adults (intestine, gills, gonads, head-kidney or mid-kidney) and from early developmental stages. The samples from different developmental stages comprised of embryos sampled at 4, 19, 39, and 50 days post fertilization (dpf), an eyed-egg 63dpf, and an alevin one-day post hatching (alevin 1dph). These 111 samples were used in the miRdeep2 analysis (version 0.0.7) [4], the analysis of tissue enriched miRNAs and for isomiR characterization (Table S1). Results from the eleven small-RNA sequenced samples in Andreassen et al. [9] were also included in the datasets to identify miRNAs enriched in particular tissues. An additional 24 tissue samples (Table S2) from brain (n = 4), gills (n = 3), heart (n = 4), intestine (n = 5), liver (n = 5) and white muscle (n = 3) were included to investigate miRNAs enriched in those particular tissues. These samples were used for RT-qPCR analysis as described in Section 2.9. 

Fry materials, sampled in Scotland were euthanized using a procedure specifically listed on the appropriate Home Office (UK) license, and all experiments were performed under the approval of Cefas ethical review committee and complied with the Animals Scientific Procedures Act. Some of the pathogens challenged fry were part of a challenge study using infectious pancreatic necrosis virus (IPNV) described in in Robledo et al. [23]. Sacrifice procedure of the other fish in the materials sampled in Norway was approved by the official ethics board FOTS (forsøksdyrutvalgets tilsyns-og søknadssystem). Dissection and sampling of materials were performed in agreement with the provisions enforced by the Norwegian Animal Research Authority.

### 2.2. Small RNA Extraction

Sampling and extraction of RNA from the fry materials (samples 1–96, Table S1) were carried out, as described in Robledo et al. [23]. Total RNA was isolated using TRI reagent (Sigma–Aldrich^®^, St. Louis, MO, USA) following the manufacturer’s instructions. The RNA quantity and quality were determined using spectrophotometry (NanoDrop ND-1000, Thermo Scientific, Wilmington, DE, USA) and agarose gel electrophoresis, respectively. Total RNA from the remaining materials was extracted by using the mirVana Isolation Kit (Ambion, Life Technologies, Carlsbad, CA, USA) according to the manufacturer’s protocol. The RNA concentration and purity were determined using spectrophotometry (NanoDrop ND-1000, Thermo Scientific, Wilmington, DE, USA). 

### 2.3. Library Preparation and Small-RNA Sequencing

Small-RNA library construction and the small-RNA sequencing was performed at the Norwegian Genomics Consortium’s genomic core facility (NGC). The Illumina NEBNext small RNA Library Prep Set (New England Biolabs, Inc. Ipswich, MA, USA) was used to prepare the 96 libraries from the 96 fry samples, while Illumina^®^ TruSeq Small RNA sample preparation kit (Illumina, San Diego, CA, USA) was used to construct 15 libraries from the tissue and developmental stage specific samples. 1 µg of total RNA was used per sample as input in the library preparation in accordance with the manufacturer’s protocols. The sequencing was carried out on the Illumina Genome Analyzer IIx sequencing platform. 

### 2.4. Pre-Processing and Quality Assesment of Small-RNA Sequence Reads

The raw sequencing reads (fastq-files) from each sample were pre-processed to ensure that the raw data used in downstream analysis were of good quality and desired sizes. An assessment of the raw sequence reads was first carried out using FASTQC (v.0.11.5) [24], to ensure that the small RNA read quality was satisfactory before adaptor sequences were removed using cutadapt (v.1.13) [25]. Additional size filtering was carried out to discard reads that were outside the expected size range of mature miRNAs (18–25 nts). The quality of the trimmed and size filtered reads were analyzed by a second FASTQC analysis. Reads that passed this post-trim QC were used in the downstream analysis of the small RNA sequenced samples. 

### 2.5. Identification of Atlantic Salmon miRNA Precursors, Their Mature miRNAs and miRNA Gene Locations 

**Figure 1:**
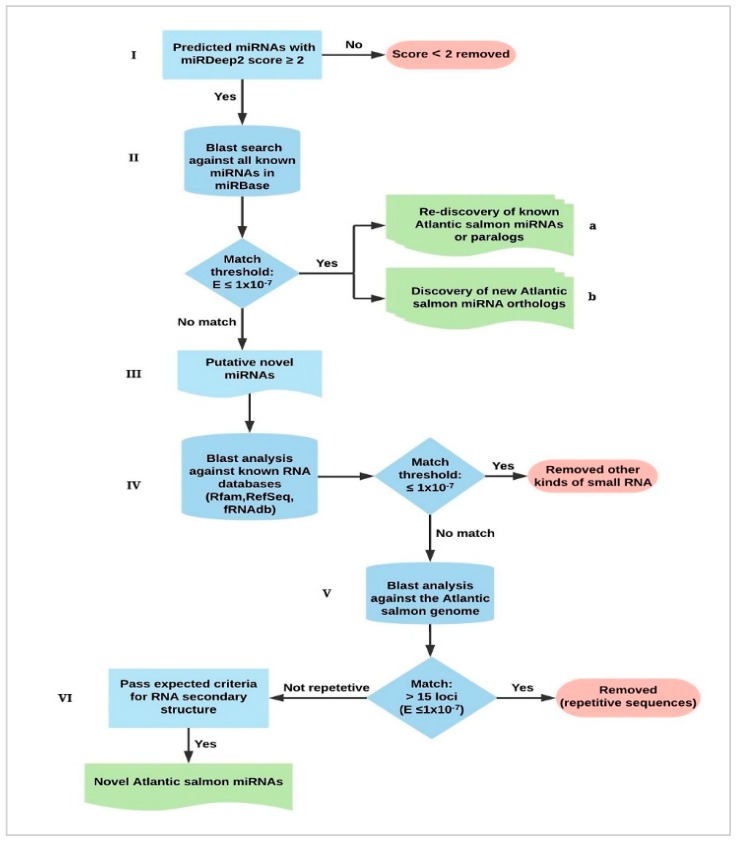
Identification and characterization of Atlantic salmon microRNAs (miRNAs). (I) Candidate miRNAs with miRdeep2 score ≥ 2 were regarded as putative miRNAs. (II) miRNA precursors were used as input for BLAST search against miRBase to identify; (**a**) Atlantic salmon miRNAs already in the database and new paralogs, (**b**) Orthologues of other teleost miRNAs not discovered in prior studies of Atlantic salmon. (III) Candidate miRNAs with no significant matches (e ≤ 1 × 10^−7^) to miRBase were considered as putative novel Atlantic salmon miRNAs. (IV) Putative novel miRNA precursor sequences were BLAST analyzed against other RNA databases (Rfam, NCBI RefSeq and fRNAdb) to remove those that were other kinds of small RNA. (V) Sequences with no matches in these databases were further used for BLAST analysis against the Atlantic salmon reference genome sequence. Significant matches (e ≤ 1 × 10^−7^) to more than 15 loci in the Atlantic salmon genome were annotated as interspersed repeats and removed. (VI) The remaining putative novel miRNAs were compared against expected features of precursors and their mature miRNAs. The candidates that passed all these criteria were regarded as novel Atlantic salmon miRNAs.

High quality reads from 108 samples (samples 1–97, 99, 102–111, Table S1) were used for identification of miRNAs applying the miRDeep2 software package. Default settings were used [3,4] to predict miRNA precursors along with their 5p and 3p mature miRNAs. Each sample was independently analyzed with miRDeep2 to allow for detection of miRNAs expressed in particular tissues or developmental stages. The present version of the Atlantic salmon genome assembly, ICSASG_v2, GenBank accession number: GCA_000233375.4 [22], and a genome index consisting of the existing reference Atlantic salmon miRNAs [9,10] was also used in the miRdeep2 analysis. The characterization pipeline is illustrated in Figure 1. The reads were mapped to the Atlantic salmon genome sequence using the miRDeep2 mapper module. Guided by the mapped reads genomic sequences that showed features expected from precursor sequences (e.g., ability to form hairpins) and with aligned reads showing expected characteristics of mature miRNAs processed by Dicer/Drosha were identified. These were assigned a log-odds score (the miRDeep2 score) based on an algorithm that integrates the statistics of the read positions, the frequencies of reads within hairpins, and the posterior probability that the hairpin was derived from a true miRNA gene [3]. To prevent false positive detection of miRNA precursors, a miRDeep2 score of ≥ 2 was used as a cut-off. All predicted precursors with miRDeep2 scores equal to or above the threshold were included in the downstream analysis. They were further analyzed by Basic Local Alignment Search Tool (BLAST) searches, using the putative precursor sequences as input, against miRNAs in the miRNA sequence database (miRBase) (version 22) (http://www.mirbase.org/search.shtml) [26]. A significant hit was defined as matches with an e-value of ≤ 1 × 10^−7^ against any hairpin precursor in the database. Those identified as Atlantic salmon miRNA genes in the first study [9] were annotated with new genome locations in the present Atlantic salmon genome. Other matches were identified as Atlantic salmon orthologs of miRNA genes discovered in other teleost species. These were annotated according to the miRBase nomenclature guidelines (ssa-prefix and same number as in other teleosts) [27,28]. The precursors that did not provide significant matches with any of the miRNAs in miRBase were potentially novel miRNAs. These were further analyzed by blastn (http://blast.ncbi.nlm.nih.gov/Blast) searches against RNA databases in GenBank (http://blast.ncbi.nlm.nih.gov/Blast), the small RNA databases Rfam v.13.0. (http://rfam.xfam.org/search) [29], and the functional RNA database fRNAdb v.3.0. (https://dbarchive.biosciencedbc.jp/blast) [30]. Candidates that matched other kinds of small RNAs in these databases were removed (e-value threshold of ≤ 1 × 10^−7^). The remaining precursors were used as queries in blastn analysis against the Atlantic salmon genome sequence. Any putative precursor that provided a significant hit (e-value threshold of ≤ 1 × 10^−7^) against more than 15 loci in the genome reference sequence were annotated as interspersed repeats and removed. RNA secondary structure of the remaining miRNA precursors and their aligned reads were manually inspected. Novel miRNA genes were identified based on passing the following miRBase criteria [26]; (1) detected in at least two independent samples, (2) at least 10 sequence reads of mature and star miRNAs mapped (with no mismatches) to the hairpin precursor, (3) the mature microRNAs were paired with the precursor hairpin with 0–4 nt overhang at their 3’ ends, (4) the reads mapped supported a consistent pre-processing of 5’ end (5’ homogeneity) and (5) more than 60% of the bases of the mature sequences paired in the hairpin structure. Those that passed all these criteria were annotated as novel Atlantic salmon miRNAs. These were designated “ssa-miR-novel-x” with identifying numbers (x; e.g., ssa-mir-novel-1). All miRNAs, including the novel ones discovered in this study have been submitted to miRBase. When assigned unique miRNA identities by miRBase these will be added as Addendum to this paper.

### 2.6. Annotation of Clustered miRNA Genes

The genome location of all Atlantic salmon miRNA genes was identified using the second and improved version of the Atlantic salmon genome assembly. The amount of clustered miRNA genes in the Atlantic salmon genome was examined by comparing their locations in ICSASG_v2. Any miRNA genes located in the same direction in a contig, within a distance of 10 kb or less were annotated as clustered miRNA genes (same definition as used by miRBase). We also compared the miRNA clusters from our study to miRNA clusters in zebrafish (*Danio rerio*) and Atlantic cod (*Gadus morhua*), as given in miRBase to reveal evolutionarily conserved miRNA gene clusters.

### 2.7. Sequence Errors Arising in the Sequencing Pipeline and IsomiR Detection

Prior to characterizing the abundance of isomiRs the amount and type of reads with erroneous sequences (erroneous sequence variants, ESVs) that arise in the sequencing pipeline (extraction, cDNA synthesis, library prep, small RNA sequencing) must be assessed. High quality reads from 48 fry samples were pooled (441,320,712 reads with Phred quality score above 32). This dataset was collapsed into a total of 7,004,792 unique reads annotated with their read count number. The phiX Control is commonly used as an internal control to measure the proportion of ESVs when larger fragments are sequenced [31,32]. This control can however, not be applied in small RNA sequencing. Instead, we used two highly abundant and ubiquitously expressed Atlantic salmon RNAs; (18S rRNA (Genbank accession: FJ710886.1), and 60S ribosomal protein L37 (GenBank accession: BT058368.1)) for this purpose. The two RNAs, the rRNA (1750 bp) and the short mRNA (540 bp), were used as references as reads that are derived from anywhere out of these larger sequences are not expected to be RNA edited. All reads were aligned against these two references. The aligned reads with bp differences compared to the references would be ESVs (or polymorphism). By aligning the unique sequence reads to those reference sequences in Sequencher software version 5.4.6 (Gene Codes Corporation, Ann Arbor, MI, USA), the ESVs were visualized. Reads that aligned perfectly to the references and reads that represented reads with ESVs were counted. Any bias in error rate within reads (e.g., higher error rate in 3’ end) was also assessed. The ratio between perfectly aligned reads and ESVs were used to set an error threshold. Reads present in lower amount than this threshold and with non-templated sequence variation compared to the canonical reference miRNAs are likely to be ESVs, not isomiR variants that are products of RNA editing. 

Detection of isomiRs was carried out by use of isomiR Seed Extension Aligner (isomiR-SEA) software (version 1.60) [33]. The tool detects mature miRNAs and mature miRNA isoforms by a seed-based alignment procedure. The unique reads were aligned to reference miRNAs (mature canonical miRNAs identified in this study) using default settings and commands. The tool classifies miRNA variations into four categories with respect to the reference miRNA sequence. The reads matching perfectly to the reference miRNA (canonical miRNAs), reads with any nucleotide variation in the 5’ end (5’ isomiRs), reads with any nucleotide variation in the 3’ end (3’ isomiRs and 3’ length isomiRs), and reads with mismatches anywhere in their sequences. Count number of the reads identical to canonical miRNA sequences was used to set a lower threshold based on the error ratios revealed in alignments to the reference sequences. If below this threshold, they were removed assuming they were erroneous sequence variants (ESVs) that had arisen in the pipeline, not isomiRs. All reads variants passing the error rate cut-off filtering were aligned to their reference miRNA sequences using Sequencher software. Degraded miRNAs would be identical, but shorter than reference miRNAs. Shorter 5’ and 3’ isomiR variants could therefore not be reliably identified. The total RNA extracted also consists of precursor miRNAs, occasionally present in a larger amount than the mature miRNAs derived from the precursor [34]. Any templated 3’ length isomiRs larger in size than the reference miRNAs could therefore not be reliably detected as such reads could arise from degradation of precursor molecules. These shorter and longer template sequences were excluded from further analysis. The isomiR variants that could be reliably detected in small RNA sequencing data sets were therefore non-templated 5’ isomiRs, non-templated 3’ isomiRs and non-templated 3’ length isomiRs. The identification of miRNAs (Section 2.4) is based on the alignment of reads to the genome sequence. Only reads that aligned perfectly would identify a miRNA gene. As ESVs would be approximately one fifth of all reads aligning, and those not aligning perfectly, they would not interfere with detection of a miRNA gene and it‘s templated, canonical mature miRNAs (reads aligning perfectly to the template).

### 2.8. Examinating the Biological Effect on Target Gene Specificity from IsomiR Variation

Target gene predictions of isomiRs and their corresponding canonical mature miRNAs was carried out using the target gene prediction software RNAhybrid version 2.2 [35]. The mature miRNAs (canonical and isomiR variants) were tested against 3’UTRs from all Atlantic salmon mRNA transcripts in the Refseq database of Genbank (https://www.ncbi.nlm.nih.gov/refseq/). The analysis was performed applying the following conditions: Helix constraint 2–8 and no G:U in seed. This allowed only target genes with perfect seed complementarity to be detected. Minimum free energy threshold for RNA hybrids was set to −18 kcal/mol to retrieve results (target site matches) from RNA hybrids with high stability. 

### 2.9. Identification of Tissue Enriched miRNAs 

The high-quality reads from 23 tissue samples (samples 6, 7, 97, 98, 100, 102, 104, 106–111 (Table S1) and the 10 samples sequenced in a previous study [9]) were used to provide rough estimations of the expression of individual miRNAs across the different tissues. Each mature miRNA was counted using the reference miRNAome and STAR aligner (version 2.5.2b) [36]. The number of miRNAs in each sample was normalized by reads per million scaling factor (RPM) [37]. In cases where there were biological replicates of same tissue (liver, spleen, kidney, head-kidney and intestine) we used the average RPM values for these tissues. The normalized read counts of individual miRNAs within each tissue were compared to identify miRNAs that were highly expressed. The fifteen most abundant miRNAs in each of the tissues, a total of 43 miRNAs, were identified in this manner. The RPM values from these 43 miRNAs were then compared to identify those miRNAs highly expressed across all tissues (three-fold or less difference) and those highly expressed in one or a few particular tissues (more than ten-fold increase in one or a few tissues). Additional tissue enriched miRNAs that were not among the fifteen most abundant miRNAs from a tissue, were identified in the same manner (RPM comparison). These were also analyzed by RT-qPCR to validate they were enriched in particular tissues (see Section 2.9). Those that showed more than ten times higher expression in a particular tissue (measured by RPM and RT-qPCR or RPM only) was termed highly expressed or tissue enriched.

### 2.10. RT-qPCR Analysis of Tissue Enriched miRNAs

Nineteen miRNAs (ssa-miR-9a/b-5p, ssa-miR-153a-3p, ssa-miR-122-5p, ssa-miR-499a-5p, ssa-miR-194b-5p and ssa-miR-192a-5p, ssa-miR-96, ssa-miR-129, ssa-miR-132, ssa-miR-135c, ssa-miR-212, ssa-miR-219, ssa-miR-723, ssa-miR-734a, ssa-miR-8163, ssa-miR-736, ssa-miR-459 and ssa-miR-140) were analyzed by RT-qPCR as their RPM values indicated high enrichment in one or few particular tissues. A summary of the samples used for verification of tissue enriched miRNA expression by RT-qPCR, a total of 24 samples, is given in Table S2. 

First strand cDNA synthesis was performed using the miScript II RT kit (Qiagen, Hilden, Germany) according to the manufacturer’s instructions. miRNAs were then detected using the miScript SYBR^®^ Green PCR kit (Qiagen, Hilden, Germany) as described by the manufacturer. The qPCR assays were carried out by means of custom designed forward primers together with a universal reverse primer provided with the miScript qPCR kit. An overview of the primers specific to each mature miRNA is given in Table S3. The qPCR reactions were performed on Mx3000p qPCR system (Stratagene, Agilent Technologies, LA Jolla, CA, USA) using 96-well plates, with thermocycling conditions of 95 °C for 15 min, followed by 40 cycles of 94 °C for 15 s, 55 °C for 30 s and 70 °C for 30 s. Cq values were obtained using the MX3000p software package (Stratagene, Agilent Technologies, USA). The relative expression of each miRNA was normalized against miR-25-3p and miR-107-3p, based on their good stability across tissues [10]. The mean normalized Cq values for each of the different tissues was calculated. The relative difference in expression between tissues was calculated using the ∆∆Ct-method [38]. Statistical significance of the observed relative difference in expression was tested using student’s t-test and significance levels corresponding to P-values ≤ 0.05 that were set after Bonferroni-correction based on number of tests. 

## 3. Results

### 3.1. Small RNA Sequencing and Identification of Atlantic Salmon miRNAs

#### 3.1.1. Generation of RNA Libraries and Results from Small RNA Sequencing 

Small RNA libraries were successfully generated for 111 samples from salmon fry (n = 24), pathogen challenged fry (n = 72), tissue samples (intestine, gills, gonads, head-kidney and mid-kidney) from fully developed adults (n = 9), and samples from different developmental stages; embryos sampled at 4, 19, 39, 50 dpf, eyed-egg 63dpf, and an alevin 1dph. After pre-processing the raw reads (see methods), there were a total of 656,748,326 high quality, adapter trimmed and size filtered reads from fry samples and 46,047,362 reads from the different tissues and developmental stage samples. A detailed overview of sample origin, RNA concentration, quality and read numbers are given in Table S1. 

#### 3.1.2. Results from Discovery and Characterization of Atlantic Salmon miRNAs 

The miRdeep2 algorithm has been shown to be a sensitive and reliable method for identifying miRNAs in different species [3,4]. Here it was successfully used to analyze the processed high-quality reads from each of the 111 samples separately. The results from the miRdeep2 analysis was 941 predicted miRNA precursors with their corresponding 5p and 3p mature reads. These were further processed as described in Figure 1 (see Section 2.4). Out of the 371 different Atlantic salmon miRNA genes already annotated and deposited in the database [9], all but one of each of the miRNA paralogs ssa-mir-210-1 and ssa-mir-29b-4 were re-discovered. In addition, new identical paralogs (i.e., identical precursor miRNA sequences at different genome locations) and new miRNA paralogs with small sequence differences, altogether a total of 533, were discovered. The re-discovery of these miRNAs in this new material provided additional confidence that they are true Atlantic salmon miRNAs. Another 20 miRNAs were identified as orthologues of miRNA genes in other teleosts and annotated as new Atlantic salmon miRNA genes in accordance with miRBase guidelines. Three of these miRNA genes had two copies (paralogs) in Atlantic salmon. We thus identified 556 evolutionary conserved miRNA genes with their corresponding mature 5p and 3p miRNAs (Table S4). 

The remaining precursor candidates retrieved from the miRdeep2 analysis showed very low sequence similarity to any of the miRNA genes in miRBase. Seventy-nine of these candidates provided significant matches (e-value ≤ 1 × 10^−7^) to other kinds of small RNA (e.g., rRNA, snoRNAs), and were removed. Hundred and twenty sequences provided multiple hits against the salmon genome sequence indicating they were different kinds of repetitive sequences rather than miRNA genes, and thus removed.

Finally, we applied the miRBase guidelines to identify high confidence novel miRNA genes among the remaining precursors retrieved from the miRdeep2 analysis [26]. Following these guidelines (see Section 2.4), 17 novel miRNA genes were identified. The precursor sequences from all novel miRNAs with their corresponding mature 5p and 3p sequences, annotation of arm dominance and the genome location of each precursor is given in Table 1. One of the novel miRNA genes revealed a perfect match in miRBase, (e-value ≤ 1 × 10^−18^) to a salmon fluke (*Gyrodactylus salaris*) miRNA (gsa-mir-9404). However, this precursor sequence aligned perfectly (100% identity) to the Atlantic salmon genome, while there were no matches to the *Gyrodactylus salaris* genome (GenBank accession: GCA_000715275.1) (e-value > 0.5). This one was also identified as an Atlantic salmon miRNA in Bekaert et al. (miR-new156-5p) [10]. This strongly indicates that this is an Atlantic salmon miRNA rather than originating from *G. salaris*. This miRNA was annotated as ssa-miR-novel-17 in our new reference miRNAome. One novel precursor (ssa-miR-novel-15) had several identical copies clustered at two unique genomic locations, and two novels (ssa-miR-novel-6 and ssa-miR-novel-7) had two and three identical copies respectively, while the other 14 novel miRNA genes were present as single copies.

In summary, there were 448 different miRNA genes, including 17 novel miRNAs discovered. Adding the 102 identical paralogs there were 577 miRNA genes. Their locations in the present Atlantic salmon genome is given in supplemental material (Table S4). The new mature Atlantic salmon miRNAome reference sequences originating from these genes were a total of 589 unique 5p and 3p mature miRNAs. These are given in FASTA format to be used as a reference miRNAome in miRNA expression studies in supplemental file S5. 

miRNA genes are often located in clusters [9,39,40,41] that may be co-transcribed as a single pri-miRNA [2]. Furthermore, miRNA genes located in the same cluster have been shown to work together to control the same gene pathways when regulating various cellular processes [42,43,44]. With the discovery of more than one hundred new miRNAs and new genome annotation of all miRNA genes, the amount of clustered miRNA genes was re-examined. miRNA gene clusters were defined as suggested by miRBase as two or more miRNA genes located less than 10 kb from each other in the same direction (on the same genomic strand) [45]. Applying this definition, we identified 235 miRNA genes that were grouped into 93 distinct clusters. These clusters account for 40% of all Atlantic salmon miRNA genes. All gene clusters, the miRNA genes in the cluster and their genomic locations are listed in Table S6.

Only six gene clusters were present in single copies (1–6, Table S6). The remaining clusters could be further subdivided into groups where each gene cluster within a group were paralogous copies. There were 23 such groups, including one miRNA gene cluster with a novel miRNA gene (ssa-mir-novel-15, group XXIII, Table S6). In the previous discovery of Atlantic salmon miRNA genes, Andreassen et al. reported a total of 84 miRNA gene clusters [9]. All these, as well as nine additional clusters were revealed in the new genome sequence. A simple comparison of all groups of Atlantic salmon miRNA gene clusters to the orthologue miRNA gene clusters discovered in zebrafish and Atlantic cod (miRBase release 22) showed that 86% of these gene clusters were present in Atlantic cod with at least one copy, while 90% of these were present in zebrafish with at least one copy. Those present in zebrafish and Atlantic cod are also shown in Table S6. The large percentage of orthologous gene clusters in these three species indicates that the majority of gene clusters are evolutionarily conserved in teleosts. In general, most of the evolutionarily conserved miRNA gene clusters showed a higher copy number in Atlantic salmon compared to zebrafish and Atlantic cod. This is in agreement with findings that miRNA genes have been retained in the Atlantic salmon genome in the process of re-diploidization from the evolutionary recent salmonid specific genome duplication [9,46].

### 3.2. Characterization of IsomiRs and Polymorphics miRNAs in Atlantic Salmon

#### 3.2.1. Sequence Errors Arising in the Sequencing Pipeline

**Figure 2:**
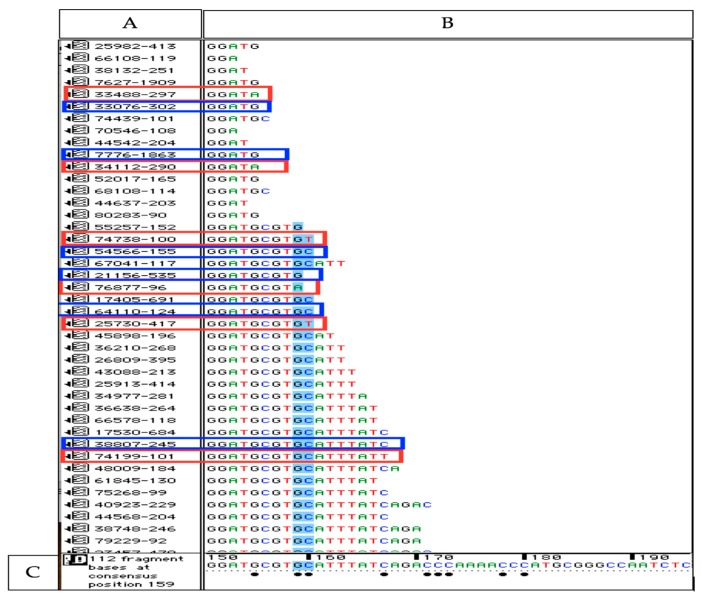
Illustration of aligned reads to the reference (18S rRNA) applying Sequencher software. (**A**) This window shows each collapsed read identified by a unique number followed by the count numbers of this particular read. (**B**) The sequence of each collapsed read is given in this window (**C**) The sequence at the bottom is the reference sequence (18S rRNA). Bullets below bases of the reference indicate that some of the reads differ from the reference at these basepair positions (erroneous sequence variants, ESVs). All collapsed reads shown in red boxes have 3’ ESVs. The collapsed reads shown in blue boxes are reads with correct bp in their 3’ end position. The ratio of reads with 3’ ESVs compared to reads with correct 3’ end bp’s in this short part of the sequence (bp 150–bp 170) was 0.10. The other reads below 3’ ESV were all identical to reference at these base positions (e.g., bases in blue color) verifying that the misaligned bases are 3’ ESVs not polymorphic variants.

The characterization of isomiRs requires that the proportion of reads with ESVs that arise in the sequencing pipeline is measured. A threshold that assures that ESVs are not incorrectly reported as isomiRs can then be set. Alignment of reads to the references (see Section 2.6) allowed us to examine the number of sequence errors arising in all parts of the sequencing pipeline. Figure 2 illustrates the results from the alignment of reads against a small part (bp 150–170) of the reference 18S rRNA. 

This revealed the amount of ESVs, as well as the position of the ESVs within reads. The ratio of ESVs distributed across any position within the reads were 0.0004. This was approximately as expected from the Phred quality of reads as a Phred score of 32 means there are less than 0.00063 bp errors due to sequencing alone. However, the alignments revealed that there was a strong bias in ESV position across reads. Almost all ESVs were positioned in the 3’ end (independent of read length). The average ratio of ESVs in these positions (last bases at 3’ end) was 0.21. The most 5’ bases also revealed a higher ratio of ESVs (0.02), but far from the ESV frequency observed at the 3’ end. This showed that other steps in the sequencing pipeline than the sequencing itself, e.g., the cDNA synthesis [19], generated a large amount of ESVs. If not accounted for, these ESVs could be misinterpreted as 3’ or 5’ isomiR variants. Alignment of reads to the two controls also showed that all different sizes of reads (18–25 nts) identical to the references were present in large numbers in the data sets. This showed, as expected, that any characterization of templated size variants (e.g., from imprecise Dicer processing) of canonical miRNAs could not be carried out as they could not be distinguished from those reads with small size variations that were just products of the pipeline itself (e.g., degradation).

#### 3.2.2. IsomiR Characterization

After a series of filtering steps and accounting for the ratio of ESVs (see Section 3.2.1), we identified 41 isomiR variants derived from 37 mature miRNAs, including four isomiR variants of novel miRNAs. Thirty-two of these were non-templated 3’ isomiRs, while eight were non-templated isomiR length variants (3’ length isomiRs). One non-templated 5’ isomiR was also identified (Table S7). The most common variants observed were, thus, the 3’ end variants (98%). Analysis of the ratio of isomiR variants vs. their canonical forms showed, in general, the predominance of the canonical mature miRNAs (Table S7). However, ten isomiR variants that were more abundant than their respective canonical forms were also observed. The most prominent change of non-templated nucleotides was from cytosine (C) to uracil (U). All eight of the 3’ length isomiRs had uracil (U) added in their 3’ ends. Such uridylation is a common RNA modification found for different RNA species, including miRNAs [47,48]. These findings indicated that the editing of mature miRNAs was not random.

**Figure 3:**
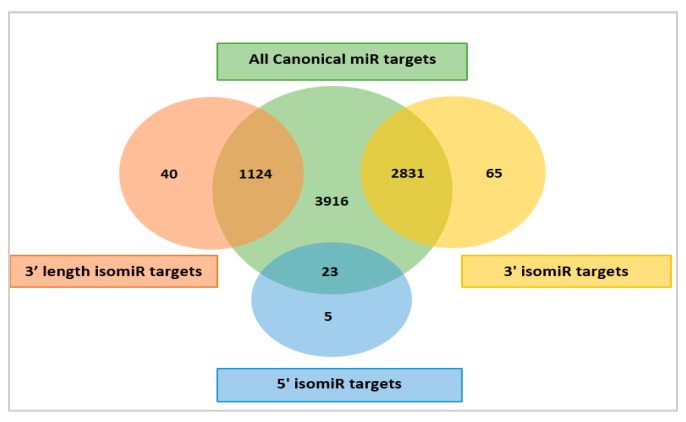
A Venn diagram depicting the overlap and difference between the predicted target genes of canonical miRNAs (green color), 3’ isomiRs (yellow color), 3’ length isomiRs (red color), and 5’ isomiRs (blue color).

The biological significance of these isomiRs was further investigated by predicting the targets of canonical miRNAs and their isomiR forms using RNAhybrid [31]. All the thirty-seven mature miRNAs and their isomiRs were analyzed against the 3’UTRs of Atlantic salmon mRNAs (Refseq, Genbank). The results (Figure 3 and Table S8) showed that the 37 canonical miRNAs together could putatively target 3916 transcripts.

The 3’ isomiRs (yellow color, Figure 3) were predicted to share 2831 common targets with their canonical miRNAs. The 3’ length isomiRs (red color, Figure 3) were predicted to share 1124 targets with their canonical miRNAs, while the 5’ isomiRs (blue color, Figure 3) were predicted to share 23 targets with their canonical miRNAs. Furthermore, the 3’ isomiRs were also predicted to target additional 65 transcripts, the 3’ length isomiRs could target 40 additional transcripts, while the 5’ isomiRs could target 5 additional transcripts not predicted as targets of their canonical forms. These comparisons showed that the isomiR variants did not lead to a large increase in putative target transcripts. Only 110 new transcripts (<3%) were added as targets when including the additional transcripts targeted by all isomiR variants. Thus, the biological effect, measured as the increase in the number of targets was small.

#### 3.2.3. Polymorphic Mature miRNAs

**Figure 4:**
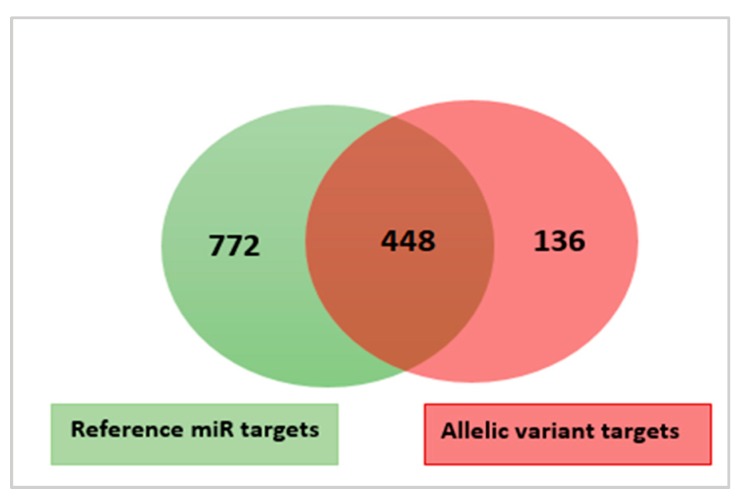
A Venn diagram illustrating the overlap and difference between the predicted target genes of the five reference mature miRNAs (green color) and the new mature miRNA variants identified in this study (light red color).

Five polymorphic miRNAs (allelic variants) were also identified (Table 2). In all cases, the proportion of reads with the new variant was larger than 40%. In one of the variants (ssa-miR-100a-2-3p), the polymorphism (T to C transition) was in the seed sequence. The biological significance of the variants was, similarly as with isomiRs, investigated by prediction of putative targets of both the reference and the new variants. Figure 4 and Table S9 show the results from this analysis. 

As shown in Figure 4, the reference mature miRNAs were predicted to target 722 transcripts. The new variants and their reference miRNAs were predicted to share 488 common targets. One hundred-thirty-six transcripts were predicted to be targeted by the new variants (increase of more than 15%) (Table S9a). The effect of allelic variation on target diversity was, thus, larger than the effect observed for isomiRs. However, this was mostly caused by the one variant where the polymorphic site was within the seed. This ssa-miR-100a-2-3p seed variant contributed 81 (60%) of all the additional targets (Table S9b). The reference mature and the new seed variant of the ssa-miR-100a-2-3p were in this case predicted to share only six common target transcripts, but mostly at different target sites on the same transcript. This indicated that a single base change in seed can significantly affect target gene specificity.

### 3.3. Characterization of miRNA Expression Profiles in Different Tissues and Developmental Stages

#### 3.3.1. Housekeeping miRNAs vs. miRNAs Predominantly Expressed in Particular Tissues

Several studies have demonstrated that miRNAs play an important role in tissue development and/or in the maintenance of tissue specific functions [5,40,49,50,51,52,53,54]. Such miRNAs are often highly expressed in one or a few tissues. On the other hand, there are miRNAs with ubiquitous high expression across most tissues assumed to maintain housekeeping functions [54]. To identify miRNAs that are likely to have such housekeeping functions the fifteen most abundant miRNAs in each of the tissues brain, liver, heart, head-kidney, muscle, intestine, kidney, spleen, gonads and gills were revealed. Together, there were 43 such miRNAs. Twenty-one of these did not reveal any large expression differences when comparing across tissues (three-fold or less). Further examining the enrichment patterns of these 21 miRNAs showed that seven of these miRNAs were among the top ten most abundant miRNAs in all tissues. These were miR-143-3p, miR-181a-3p, miR-21b-5p, mir-26a-5p, miR-10b-5p, mir-10d-5p and mir-10a-5p (Table S10). Together, these seven miRNAs accounted for more than 30% of all miRNAs expressed in any tissue. Their ubiquitous nature and high expression in many or all tissues suggest that these miRNAs are constitutively expressed and have housekeeping functions.

**Figure 5:**
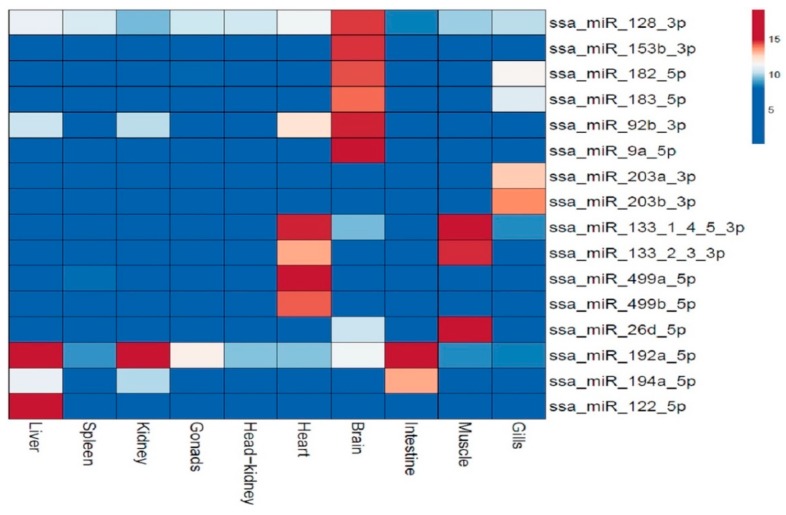
The heatmap illustrates the predominant expression of 16 mature miRNAs in particular tissues. The tissues compared are given at the bottom of the columns, while the miRNAs are given in the horizontal rows to the right. Four of these miRNAs (ssa-miR-9a-5p, ssa-miR-499a-5p, ssa-miR-192a-5p and ssa-miR-122-5p) were also analyzed by RT-qPCR (see Table 2). The expression values (log2) of the miRNAs are depicted in the color scale. Very little or no expression is shown in dark blue, while other colors indicate increased expression (10–100 times) with dark red color as those with the higher expression.

Other miRNAs showed a predominant expression in particular tissues. Although they were among the 15 most highly expressed miRNAs in one tissue, they showed very little or no expression in the other tissues. All of these showed 10–100 times higher expression in one or few particular tissues. These are shown in the heat map in Figure 5.

Six of these miRNAs (ssa-miR-128-3p, ssa-miR-153b-3p, ssa-miR-182-5p, ssa-miR-183-5p, ssa-miR-92b-3p and ssa-miR-9a-5p), were predominantly expressed in brain. Two miRNAs from the miR-499-family (ssa-miR-499a/b-5p) showed about 70 times higher expression in cardiac tissue, while all 3p mature miRNAs in the miR-133 family (ssa-miR-133-1-4-5/2-3-3p) showed higher expression in both muscle and cardiac tissue. Another miRNA that was predominantly expressed in muscle tissue was ssa-miR-26d-5p (about 20 times increase). Two miRNAs, ssa-miR-203a/b-3p, were highly expressed in gills. Ssa-miR-192a-5p was predominantly expressed in intestine, but this miRNA also showed an eight to twenty times higher expression in kidney and liver than in all other tissues indicating it was serving a function common to a cell type present in these three tissues. One more miRNA, ssa-miR-122-5p also showed a predominant expression in the liver.

Another 15 miRNAs showed lower abundance (i.e., not among the 15 most abundant miRNAs in one or more tissues), but were still enriched in specific tissues. Additional measurements by RT-qPCR were used to show that there was a significantly higher expression (>10× higher) of these in the particular tissues (*p*-adjusted ≤ 0.05). The results from RT-qPCR analysis of all these miRNAs (Table 3) agreed with the patterns revealed by the RPM comparisons.

A complete overview of all 31 miRNAs that showed a tissue specific expression pattern is given in Table 4. Brain tissue was the one tissue showing largest number of miRNAs expressed in a tissue specific manner, both among those miRNAs with highly enriched expression (Figure 4), as well as those investigated by RT-qPCR (16 miRNAs, Table 4).

#### 3.3.2. Expression Patterns of miRNAs at Development Specific Stages

**Figure 6:**
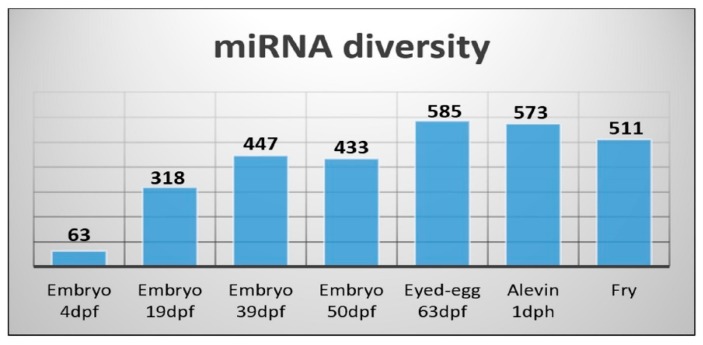
miRNA diversity measured as a number of different miRNAs detected across developmental stages.

It has been reported that miRNAs show developmental stage-specific abundance during the embryonic development of teleosts [32,37]. The miRNA diversity showed, in general, an increase from less than one hundred to close to 600 different miRNAs during development. Embryo 4dpf, corresponding to the earliest developmental stage in our materials, showed the smallest diversity with only 63 different miRNAs expressed (Figure 6). There was a very large increase in the number of miRNAs that were expressed during the next 15 days as the embryo 19dpf sample revealed there were 318 different miRNAs expressed. The number of miRNAs detected increased by another hundred the next 20 days (embryo 39dpf), and at the eyed-egg stage (63dpf) it was at its maximum (585 different miRNAs).

**Figure 7:**
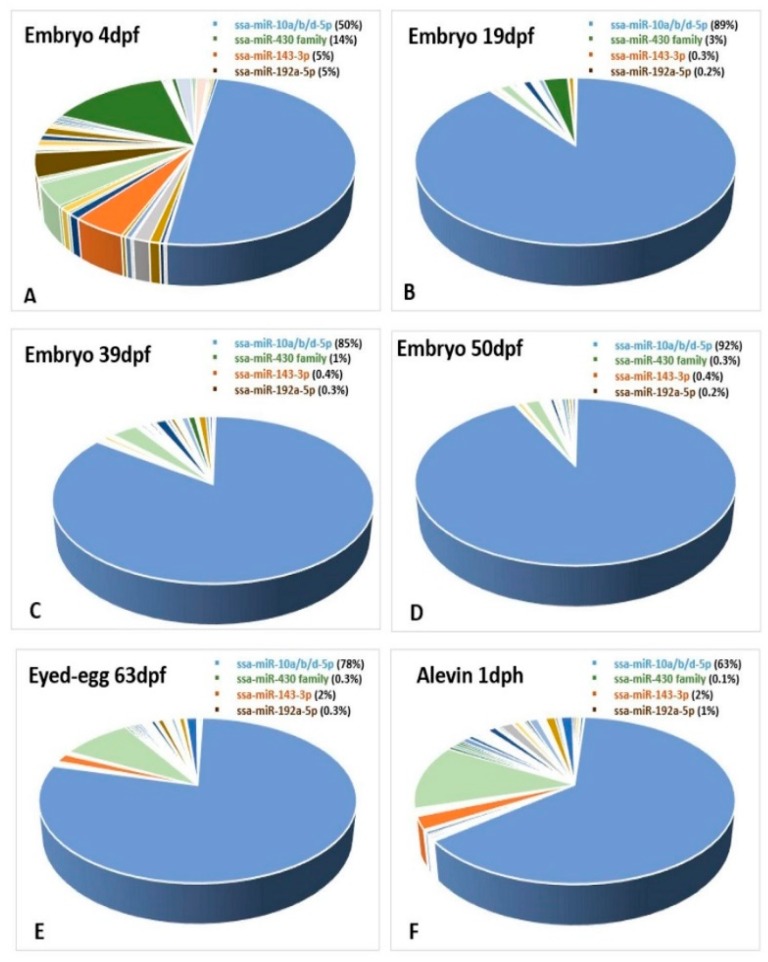
Abundance as a proportion of the total number of miRNAs within each of the developmental stages. The ssa-miR-10a/b/d-5p family, the miR-430 family, ssa-miR-143-3p and ssa-miR-192a-5p are shown in blue, green, orange and brown colors, respectively, in the figures (**A**–**F**).

**Figure 8:**
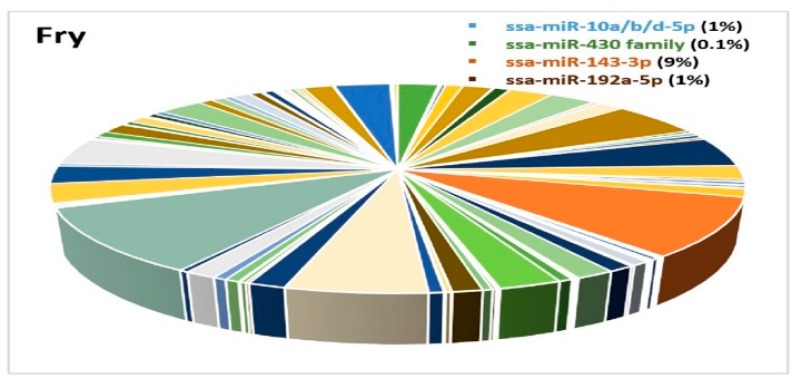
A pie chart demonstrating the miRNA expression diversity (511 miRNAs) in fry. The ssa-miR-10a/b/d-5p family, the miR-430 family, ssa-miR-143-3p and ssa-miR-192a-5p are shown in blue, green, orange and brown colors, respectively, in Figure 8.

Due to the limited number of samples and lack of proper normalization, we could not compare expression differences between the developmental stages. We could however compare the expression differences of miRNAs within each of the developmental stages. A few miRNAs showed high expression at specific stages, while others exhibited a ubiquitous expression pattern and were highly enriched in all the developmental stages. This was the case for the three members of the mir-10 family (ssa-miR-10b-5p, ssa-miR-10d-5p and ssa-miR-10a-5p) that constituted a large proportion (>50%) of all miRNAs at most developmental stages (Figure 7A–F), while their abundance was very low in fry (1% of all miRNAs) (Figure 8). The proportion of miRNAs from the ssa-miR-430 cluster was relatively large at the earliest stages of development (embryo 4dpf, Figure 7A), but appeared to decline rapidly as the proportion was 3% at 19dpf and less than 1% at all other stages (Figure 7B–F and Figure 8).

The proportion of ssa-miR-143-3p and ssa-miR-192 was about 5% in embryo 4dpf (Figure 7A), but the proportion of these miRNAs declined about a tenfold in the following stages (19dpf to eyed-egg 63dpf, Figure 7B–E). These two miRNAs seemed to increase their expression post hatching (Figure 8).

## 4. Discussion

### 4.1. Small RNA Sequencing and Identification of Atlantic Salmon miRNAs

In this study, we performed a comprehensive analysis for miRNA characterization and identified 448 different miRNA genes in Atlantic salmon, including 17 novel miRNAs. Although a high number of Atlantic salmon miRNAs had been identified in 2013, nearly one hundred new miRNA genes, both miRNAs conserved in teleosts, as well as novel miRNAs, were discovered in this study. The increase in number of miRNA genes discovered could largely be attributed to the large number of materials included (111 samples from different tissues, developmental stages, fry and pathogen challenged fry). This miRNAome will be the new, improved reference to apply when investigating differential miRNA expression in Atlantic salmon. Furthermore, the genomic locations of all miRNA genes and their clustering patterns annotated in the updated Atlantic salmon genome sequence will facilitate further studies of the comparative evolution of miRNA genes.

### 4.2. Characterization of IsomiRs and Polymorphics miRNAs in Atlantic Salmon

With the advancement of high throughput sequencing techniques, many recent studies have reported the presence of a number of mature miRNA sequence variants with different 5’ and/or 3’ ends compared to their corresponding canonical mature miRNAs termed as isomiRs. Small-RNA sequencing projects generate datasets consisting of millions of reads differing in length and quality. Before characterizing isomiRs one need to control size variations and bp errors that arise in the pipeline. The read alignments to the larger sized quality control references indicated that most of the size variations (variation in read length) were artificial and generated in the RNA extraction or the first steps of the sequencing pipeline. There may be some templated isomiR length variants in Atlantic salmon, but due to the platform related high proportion of read length variation, they could not be detected. While there are some studies that report that they have accounted for sequence errors when characterizing isomiRs, many either have used Phred quality score estimates as an error threshold or spike-in controls to measure the sequence errors [15,55]. Despite the fact that Phred score is a good measure of sequence quality, such estimates will only account for the errors caused by the sequencing itself, not other sources of sequence errors that could be generated at other steps of the pipeline (e.g., cDNA synthesis). A high frequency of site-specific bias (especially at the 3’ ends), that would otherwise not be identified by a Phred quality score, was revealed when we estimated the average ratio of ESVs in our data (see Section 3.2.1). As isomiRs are mature miRNA sequence variants with non-templated nucleotide differences in the 5’ or 3’ ends, a large proportion of the erroneous sequence variants revealed could potentially be mistaken for isomiRs. This illustrates the importance of incorporating measurements of error beyond Phred quality to distinguish ESVs from isomiRs. The proportion of ESVs may, however, differ between library preparation methods and sequencing platforms. Nevertheless, controlling the error rate seems crucial.

Most isomiRs identified in our study were the non-templated 3’ isomiRs in the form of nucleotide substitution and/or nucleotide addition. This result is not surprising, as the 3’ isomiR variants are the most common isomiR variants observed in animals and plants [19]. As shown in Figure 3, the isomiRs variants were predicted to cause only very small changes in the number of targeted transcripts. Our findings are consistent with those reported in other studies, as targeting is mainly mediated through complementary binding of the 5’ seed (2–8 nts of mature miRNA sequence) [17,19]. Moreover, the total amount of isomiR variants was small (43 isomiRs). Together, these findings suggest that these types of modifications may have less biological impact than anticipated [56]. Allelic variation in the seed did however have a major effect on target gene diversity. The target gene analysis of the two allelic variants of miR-100a-2-3p (Table 2) showed that the new variant practically acted as a “new miRNA”, with a completely new set of target transcripts. The negative selective pressure against such variation, that could allow miRNAs to adapt new regulatory functions, may be less strict when miRNAs are from families where the other members maintain regulation of their original target genes. As the partially tetraploid Atlantic salmon miRNAome has a much larger number of very similar miRNA genes (paralogs) than other diploid fish (e.g., zebrafish, Atlantic cod), this would allow for WGD-derived paralogs to change in seed and develop new functions.

Although the biological significance of 3’ isomiRs and 3’ length isomiRs seems small, these may still cause methodological issues when analyzing miRNAs by quantitative real time PCR (RT-qPCR) [12,16]. This miRNA detection methodology mainly depends on amplification that is initiated with a miRNA-specific primer. A nucleotide difference in the 3’ end (3’ isomiRs and 3’ length variants) could lead to the detection of products with different melting temperatures and thus, affect precise measurements of specific miRNA levels as both the canonical miRNA and the isomiR variant may be cross detected [57]. It is therefore important to be aware of such variants, as they may explain some of the methodological challenges one may come across when measuring miRNA expression by RT-qPCR [16].

### 4.3. Characterization of miRNA Expression Profiles in Different Tissues and Developmental Stages

Identifying expression patterns of Atlantic salmon miRNAs in different tissues and developmental stages provides important insight into the function of individual miRNAs. The miRNAs discovered in our study showed a wide range of expression profiles in the different tissues and developmental stages. The miRNAs ssa-miR-143-3p, ssa-miR-181a-3p, ssa-miR-21b-5p, ssa-mir-26a-5p, ssa-miR-10b-5p, ssa-mir-10d-5p and ssa-mir-10a-5p were ubiquitously expressed in all tissues tested. The high abundance and ubiquitous expression profile of most of these miRNAs have been reported in several other species [58,59,60,61]. The evolutionarily conserved high expression profiles of these miRNAs suggest they are associated with common signaling pathways in vertebrates and have the same housekeeping functions in Atlantic salmon. Other miRNAs showed a tissue specific expression pattern suggesting a specialized role for these miRNAs in tissue differentiation or maintenance of tissue specific functions. The brain-enriched miRNAs, such as the miR-9 family are e.g., known to have important roles in neurogenesis and brain development in other fish and mammals [43,53,54,58]. Also consistent with previous findings in salmon and other teleosts was the tissue specific high enrichment pattern of miR-122-5p in liver [39,51,52,62]. The high expression of miR-192 shown in liver, kidney and intestine tissues have also been reported in other fish species [39,63]. Finally, there was a high expression of the miR-133 family in both muscle and cardiac tissue, whereas the miR-499 family members were only enriched in cardiac tissue. The miR-133 family are amongst others known to regulate cardiomyocyte differentiation and proliferation, cardiac morphogenesis and stress responsive cardiac remodeling process in other species [64,65]. As demonstrated in other species, a majority of the orthologous miRNAs that showed tissue specific expression have specialized functions in different tissues. When revealing same tissue enriched expression in Atlantic salmon it is likely that they also have similar specialized functions in this species. 

Several studies have suggested that miRNAs have essential roles in the developmental progression in vertebrates [66,67]. The increase in miRNA diversity along with the different stages of development (Figure 6) indicates that the developmental processes are under miRNA regulation during Atlantic salmon development. The largest change in the proportion of miRNAs within the different developmental stages was observed for the miR-430 family members. This miRNA family were highly abundant during the earliest stages of development, while their abundance decreased rapidly throughout the later stages (Figure 7 and Figure 8). This pattern of expression agreed with those of miR-430 reported in zebrafish. It is assumed that this family of miRNAs are involved in maternal RNA clearance during early embryogenesis [49,68,69]. We also found three members of the miR-10 family that were highly abundant throughout all developmental stages up to one-day post-hatching (Figure 7). Previous studies in other teleosts have identified members of the miR-10 family members as key regulators of *Hox* genes, which are important regulators of embryonic development in vertebrates [50]. The expression of miR-10 family members is, among others, also shown to be associated with mediating cell proliferation and differentiation [70]. The sequences of these Atlantic salmon miRNA family members are highly conserved across species [70]. Together, this implies that they may have similar functions in Atlantic salmon development. The proportion of ssa-miR-143-3p and ssa-miR-192 was higher in embryo 4dpf (Figure 7A) and fry (Figure 8) (Section 3.3) compared to all the other developmental stages. The investigation of tissue specific expression showed that they have very high expression levels in all tissues (ssa-miR-143-3p) or in particular tissues (ssa-miR-192a-5p). In addition, miR-143 accounted for a large proportion (10%) in female gonads, which suggests that these two miRNAs have important functions in adults. Thus, the high abundance of these miRNAs in embryo 4dpf could reflect the maternal contribution rather than a particular function at the earliest stage of development. The rapid disappearance in the later stages could be essential to allow the expression of genes important in development. Further experimental studies are necessary to reveal the particular roles of the miRNAs that showed a specific expression in some tissues and developmental stages.

## 5. Conclusions

We discovered nearly one hundred new miRNA genes, both miRNAs conserved in teleosts, as well as novel miRNAs, thus, contributing to a major expansion in the number of different miRNAs characterized in Atlantic salmon. The resulting new reference miRNAome provides an important updated resource for miRNA expression studies. Further, a subset of miRNA genes highly abundant in one or more tissues and developmental stages were revealed, suggesting important biological functions of particular miRNAs in the maintenance of tissue specific functions and in the regulation of embryonic development. Together, results from this study provide insight on miRNA regulation that includes those biological processes, and that may be of economic importance to the aquaculture industry.

## Figures and Tables

**Table 1:** Novel Atlantic salmon miRNAs identified in this study.

miRNA ID ^1^	Mature-5p (5´-3´) ^2^	Mature-3p (5´-3´) ^2^	Precursor Sequence (5´-3´)
ssa-mir-novel-1	UCAGUGAUGUGUACGCCAAAGGU	UCGGCAUACACAUCACUGACA	UCAGUGAUGUGUACGCCAAAGGUGUAAAGCUUCAAGUUCCUCGGCAUACACAUCACUGACA
ssa-mir-novel-2	AGUUUCCCGGACACAGAUUAAGCC	UUUUGUCUGUCUGGGAAACCGG	AGUUUCCCGGACACAGAUUAAGCCUAGUCAUAAUUAUUAUGUUUUGUCUGUCUGGGAAACCGG
ssa-mir-novel-3	UGACGAUACCUUUGGAACAAGA	UUGUACCAAUAGUAAAGUCUGA	UGACGAUACCUUUGGAACAAGAGGUGAAUUACGUCUUAUGCUCUUGUACCAAUAGUAAAGUCUGA
ssa-mir-novel-4	CGGAUCGCUGCGUUCACCAUU	AUGGUGAAUGCAACGAUAAGGC	CGGAUCGCUGCGUUCACCAUUAUAUUUAACUUCAACAGAAUGGUGAAUGCAACGAUAAGGC
ssa-mir-novel-5	UACGGUAUGUACUGUAGGCUAC	UAGGCUACGGUAUGUACUGAAG	UACGGUAUGUACUGUAGGCUACGGUAUGUUAUGUACUGUAGGCUACGGUAUGUACUGAAG
ssa-mir-novel-6	UGAGCCUUGUCCUGGACUAAGA	UCAGUCCAUGACUAGGCUUAAC	UGAGCCUUGUCCUGGACUAAGAAGUACUUCCAAUGGCUAUUUUCAGUCCAUGACUAGGCUUAAC
ssa-mir-novel-7	UUGCUGGUGACACUGUCUGUGA	AAGGCACACUUCACCAGUAUGG	UUGCUGGUGACACUGUCUGUGAUUUAUUUAGAAUUCAAGGCACACUUCACCAGUAUGG
ssa-mir-novel-8	AGACACCUGACACAGCCCCCAUU	UGGGUCUGUGUCUAUUGUCUCU	AGACACCUGACACAGCCCCCAUUCUAUCUCAUAAAAGUGGGUCUGUGUCUAUUGUCUCU
ssa-mir-novel-9	UAGGCGUGUCACUGCGUGUCACA	UGCGCACGGGGCCACGCUCUGC	UAGGCGUGUCACUGCGUGUCACAGUCACUGCUUGCGCACGGGGCCACGCUCUGC
ssa-mir-novel-10	AGGUCUGUUUGUGCUGUCUUCC	GUGACUGCACAAACGGAUCUGG	AGGUCUGUUUGUGCUGUCUUCCAUGGCUUUGGUGACUGCACAAACGGAUCUGG
ssa-mir-novel-11	AUUGUUCAGGGCAUUCAUUUCU	UAAGUGAACCCUUGAGACAAUU	AUUGUUCAGGGCAUUCAUUUCUUGUGAACCAAUCAAUAAGUGAACCCUUGAGACAAUU
ssa-mir-novel-12	UUCGCCCCUGAGGACACACGGU	CCGAAUCCACAGAAGUGAUGC	UUCGCCCCUGAGGACACACGGUGUUUUCUUUUAAUAGCACCGAAUCCACAGAAGUGAUGC
ssa-mir-novel-13	CCUUGACCACGUAACCUGACCA	UUAGGUCAGAUGUGGUCAGGAGA	CCUUGACCACGUAACCUGACCAUAGUUUUCUUGGUUAGGUCAGAUGUGGUCAGGAGA
ssa-mir-novel-14	GGGAAUAUACAUGACUGUGAUU	UCACAGUCGUGUAUAUUCCCUC	GGGAAUAUACAUGACUGUGAUUAUGAUUGAAGAGAAUAAUCACAGUCGUGUAUAUUCCCUC
ssa-mir-novel-15	CAGAGCUCUGCUAUCUGCUGUCU	AAGGAGAAAACAGAGCUCUGCU	CAGAGCUCUGCUAUCUGCUGUCUGUAUCUUGUUAAAGGGGAAGGAGAAAACAGAGCUCUGCU
ssa-mir-novel-16	UUGCUGUUGACACUGUCUGUG	UCAAGGCACACUUAACCAGCAUGG	UUGCUGUUGACACUGUCUGUGAUUUAUUUAAGGCACACUUCAAGGCACACUUAACCAGCAUGG
ssa-mir-novel-17	GCGUCUCAGAGGUCAAACACAGU	UGUGUUAGGCCUCCGAGUCUGA	GCGUCUCAGAGGUCAAACACAGUAAGUCAUAUUAAGCUGUGUUAGGCCUCCGAGUCUGA

^1^ Temporary annotation for the novel miRNAs identified. These will be renamed by miRBase when uploaded in the miRNA database. ^2^ The dominant mature miRNA is given in bold. Genomic location of the novel precursors is given in Table S4.

**Table 2:** Polymorphic variants of canonical mature miRNAs.

miRNA	Reference Sequence ^1^	Variant Sequence ^2^
ssa-miR-16a-1-3p	CCAGTATTGTTCGTGCTGCTGA	CCAGTATTG**C**TCGTGCTGCTGA
ssa-miR-100a-2-3p	ACAAGCTTGTGTCTATAGGTATG	ACAAGCT**C**GTGTCTATAGGTATG
ssa-miR-2188-3p	GCTGTGTGAGGTCAGACCTATC	GCTGTGTGAGGTC**G**GACCTATC
ssa-miR-29b-1-5p	ACTGATTTCTTCTGGTGTTTAGA	ACTGATTTC**C**TCTGGTGTTTAGA
ssa-let-7a-2-3p	CTATACAACTTACTGTCTTTCC	CTATACAAC**A**TACTGTCTTTCC

^1^ The reference sequence of mature miRNA. ^2^ Variant sequence with the polymorphic base given in bold. The most common variant is underlined.

**Table 3:** Results from RT-qPCR analysis.

miRNA	Tissue ^1^	ΔΔCT ^2^	Enrichment ^3^
ssa-miR-9a-5p ^4^	B	−11.13	2241
ssa-miR-9b-3p	B	−10.21	1184
ssa-miR-96-5p	B	−5.18	36
ssa-miR-129-5p	B	−3.56	12
ssa-miR-132-5p	B	−7.46	176
ssa-miR-135c-5p	B	−7.05	133
ssa-miR-153a-3p	B	−10.08	1082
ssa-miR-212ab-3p	B	−7.29	156
ssa-miR-219a-3p	B	−7.66	202
ssa-miR-723-5p	B	−6.83	114
ssa-miR-734a-3p	B	−4.68	26
ssa-miR-122-5p^4^	L	−12.3	5043
ssa-miR-8163-3p	L	−8.6	388
ssa-miR-192a-5p ^4^	L	−7.96	249
ssa-miR-499a-5p ^4^	H	−9.7	832
ssa-miR-736-3p	H	−14.7	26616
ssa-miR-192a-5p ^4^	I	−11.9	3822
ssa-miR-459-5p	I	−12.9	7643
ssa-miR-194b-5p	I	−10.8	1783
ssa-miR-140-3p	G	−3.7	13

^1^ Tissue where the particular mature miRNA is highly expressed (B = brain, L = liver, H = heart, I = intestine, G = gills, M = muscle). ^2^ Logfold difference in highly expressed tissue relative to mean of all other. ^3^ Times increase in the highly enriched tissue.^4^ These four miRNAs are also among those shown in Figure 4. All differences were significant at adjusted *p*-values ≤ 0.05.

**Table 4:** miRNAs that show tissue enriched expression patterns.

Gills	Muscle	Intestine	Brain	Heart	Kidney	Liver
ssa-miR-140-3p	ssa-miR-133-1/2-3p	ssa-miR-192a-5p	ssa-miR-128-3p	ssa-miR-133-1/2-3p	ssa-miR-192a-5p	ssa-miR-122-5p
ssa-miR-203a/b-3p	ssa-miR-26d-5p	ssa-miR-194a/b-5p	ssa-miR-129-5p	ssa-miR-499a/b-5p		ssa-miR-192a-5p
		ssa-miR-459-5p	ssa-miR-132-5p	ssa-miR-736-3p		ssa-miR-8163-3p
			ssa-miR-135c-5p			
			ssa-miR-153a/b-3p			
			ssa-miR-182-5p			
			ssa-miR-183-5p			
			ssa-miR-212ab-3p			
			ssa-miR-219a-3p			
			ssa-miR-723-5p			
			ssa-miR-734a-3p			
			ssa-miR-92b-3p			
			ssa-miR-96-5p			
			ssa-miR-9a-5p			
			ssa-miR-9b-3p			

Overview of all mature miRNAs enriched in particular tissues. Different family members that are enriched in the same tissue are indicated by a slash (e.g., ssa-miR-449a/b-5p). Identical family members are shown together as given in the reference miRNAome (Supplementary file S5), (e.g., ssa-miR-212a-3p and ssa-miR-212b-3p are shown as ssa-miR-212ab-3p).

## Supplementary Materials

The supplementary materials are available online at http://www.mdpi.com/2073-4409/8/1/42/s1.
